# Do teaching staff trust stakeholders and tools in learning analytics? A mixed methods study

**DOI:** 10.1007/s11423-023-10229-w

**Published:** 2023-04-27

**Authors:** Asma Shannan Alzahrani, Yi-Shan Tsai, Naif Aljohani, Emma Whitelock-wainwright, Dragan Gasevic

**Affiliations:** 1 Centre for Learning Analytics, Faculty of Information Technology, Monash University, Melbourne, Australia; 2 Faculty of Computing and Information Technology, King Abdul-Aziz University, Jeddah, Saudi Arabia; 3 School of Informatics, University of Edinburgh, Edinburgh, United Kingdom

**Keywords:** Learning analytics, Teaching staff, Trust, Higher education, Mixed methods

## Abstract

Learning analytics (LA) has gained increasing attention for its potential to improve different educational aspects (e.g., students’ performance and teaching practice). The existing literature identified some factors that are associated with the adoption of LA in higher education, such as stakeholder engagement and transparency in data use. The broad literature on information systems also emphasizes the importance of trust as a critical predictor of technology adoption. However, the extent to which trust plays a role in the adoption of LA in higher education has not been examined in detail in previous research. To fill this literature gap, we conducted a mixed method (survey and interviews) study aimed to explore how much teaching staff trust LA stakeholders (e.g., higher education institutions or third-parties) and LA technology, as well as the trust factors that could hinder or enable adoption of LA. The findings show that the teaching staff had a high level of trust in the competence of higher education institutions and the usefulness of LA; however, the teaching staff had a low level of trust in third parties that are involved in LA (e.g., external technology vendors) in terms of handling privacy and ethics-related issues. They also had a low level of trust in data accuracy due to issues such as outdated data and lack of data governance. The findings have strategic implications for institutional leaders and third parties in the adoption of LA by providing recommendations to increase trust, such as, improving data accuracy, developing policies for data sharing and ownership, enhancing the consent-seeking process, and establishing data governance guidelines. Therefore, this study contributes to the literature on the adoption of LA in HEIs by integrating trust factors.

## Introduction

The education sector has become more technology-orientated, where learning and teaching environments generate large amounts of data (Romero & Ventura, 2010). Currently, the world is now undergoing a so-called ’data revolution’, where vast amounts of data are produced at great speed from various sources, which also allows higher education institutions (HEIs) to collect more data than ever before (Jacqueline Bichsel, 2017). This is enabled through the use of digital technologies in education that allow for the collection of large amounts of data about learner and teacher interactions (Castro et al., 2007) in the form of digital traces that can be harnessed in learning (LA) to produce benefits for the education sector (Khalil & Ebner, 2015).

In light of the COVID-19 pandemic, HEIs have increased their use of digital platforms and tools, which magnify opportunities for data collection and use. Data collected from these tools and the platform can be analysed using LA tools to provide learning guidance by detecting learning patterns and strategies (Gašević et al., 2015; Srivastava et al., 2022), and allow students to have a better learning experience focused on their individual needs, characteristics and goals (Siemens et al., 2013; Tsai et al., 2020; Viberg et al., 2018). With increased interest in the potential of LA comes an entirely new dilemma: Do teaching staff trust LA, and if so, why? Distrust in LA can pose a challenge in the adoption of LA. Stakeholders may become interested in using LA, but may not trust LA-based reports and, as a result, may not be willing to use such reports to inform their decision-making. Trusting in the IT’s competence means that IT is considered to have the functionality or technical potential to perform any role the trustee needs to perform (Mcknight et al., 2014). Thus, while LA might have the features required to provide relevant information or services, trust in the functionality of LA depends on the capacity of LA to properly perform various services.

Previous research in LA initially focused mainly on the technical (e.g. predictive modelling) and social (e.g., ethics, privacy, and leadership) aspects of LA (Drachsler et al., 2010; Tsai et al., 2018). Trust in LA has only recently begun to receive relatively limited attention (Jones et al., 2019). To fill this gap in the literature on trust in LA, the study reported in this paper was motivated by two research questions:

1. *To what extent do teaching staff trust LA stakeholders and LA tools?* To address this question, we used survey data to explore the perceptions of teaching staff about their level of trust towards LA tools and stakeholders.
2. *What factors shape teaching staff trust in LA stakeholders and tools?* To address this question, we used interview data to investigate the factors that impact the teaching staff’s trust in relevant LA stakeholders and tools.

## Literature review

### Definition of trust

Existing research typically defines trust by referencing dimensions such as expertise, reliability, and the possibility of benefits. For example, Grandison and Sloman (2000, p. 3) define trust as the “firm belief in the competence of an entity to act reliably and securely within a specific context”. Leveille (2006) defines trust as “the belief driven by positive expectations of outcomes based on experience and perception”[p. 87]. In other words, trust is a complex concept related to dimensions such as security, truthfulness, competence, and reliability of a trusted person or service that may have to be considered in the context in which trust is formed, especially in a competitive global context such as education. It is clear that the concept of trust is receiving increasing attention in HEIs and seeing a nascent interest in e-learning research (Simmons et al., 2012; Wang, 2014). However, trust has rarely been investigated in LA research. The results of LA may help senior managers and teaching staff make data-informed decisions that can improve educational processes. Therefore, trusting LA-related tools to make data-informed decisions is an important element in LA adoption (Egetenmeier & Hommel, 2020).

### Trust in technology

With the widespread adoption of information and communication technology (ICT) across many spores of modern society, the issue of trust in technology has become evident (Taddeo, 2010). Trust in technology is “the attitude that an agent will help achieve the goals of an individual in a situation characterised by uncertainty and vulnerability” (Lee & See, 2004, p. 54). Several studies have shown that the level of trust of users in technology affects their use of technology (Muir, 1994). In general, trust is a fundamental factor in socio-technical relationships (Montague et al., 2010). Two types of trust are essential for optimal results in social-technical systems: institutional trust—a person’s trust in an institution (Montague et al., 2010); and technological trust—a person’s trust in technology (Montague et al., 2010; Muir, 1987). Trust in technology is different from institutional trust due to the distinctive characteristics of trustees. However, trust in technology cannot substitute institutional trust but should complement it (Li et al., 2012). Muir (1987) argues that user trust in technology may affect their trust in other referents, such as management or developers. This is a critical issue for sectors such as education, in which trust between individuals and technology is crucial. Therefore, to build trust in LA, it is necessary to trust LA technology and stakeholders to use or adopt LA.

### Trust in learning analytics

Existing studies in LA focus primarily on the trust of a sub-group of stakeholders (e.g., students or teachers) (Slade et al., 2019; Taddeo, 2010) without adequately contrasting perspectives of trust in LA tools and different stakeholder groups who are involved in LA. Thus far, much research on trust has treated it as a multidimensional concept and categorised trust into several dimensions depending on the referents of trust in LA. For example, Klein et al. (2019a)discussed trust in HEIs in terms of LA adoption from the perspectives of teaching staff and professional advisory staff. Slade et al. (2019) and Jones (2019) refer to the trust of students in HEIs. Another example is Klein et al. (2019a), who consider students’ trust in predictive data, and Egetenmeier and Hommel (2020), who consider trust in the LA implementation process. Recently, ) distinguished between teaching staff’s and students’ trust in LA. Extrapolating from the above arguments, the targets of trust in LA can be categorised into two general forms of trust in LA: (i) trust in stakeholders and (ii) trust in LA tools. We discuss these two forms further in the following.

#### Trust in learning analytics stakeholders (e.g., HEIs, third-party)

Perceived trustworthiness of a party is an essential antecedent of trust (Cheung & Lee, 2000). A trustworthy university positively affects the decisions of the teaching staff to trust LA by supporting the use of LA tools (Klein et al., 2019a, 2019b, 2019c). This issue is related to the results of a survey that showed a strong trust of teaching staff in receiving guidance on accessing LA and having access to data about students in a degree programme (Tsai, Whitelock-Wainwright, et al., 2021b). Furthermore, the same study by (Tsai, Whitelock-Wainwright, et al., 2021b) showed that privacy issues related to educational data have an impact on trust, where the study results showed that teaching staff had the highest expectation that HEIs would have a policy for privacy protection and ethics in LA. In terms of trust in LA stakeholders, the current study considered trust in such stakeholders as institutions that are represented by administration and management (Cho & Park, 2011; Dzimińska et al., 2018) and third parties, such as technology vendors who provide LA services or tools to HEIs.

#### Trust in learning analytics tools

Trust in LA-related tools to make data-driven decisions is an important element in LA adoption (Egetenmeier & Hommel, 2020). However, the adoption of LA has been hampered by the lack of clear, relevant, timely, and trustworthy data (Klein et al., 2019a) , the absence of reliable technological infrastructure, and the lack of alignment and integration of LA tools and data with existing technologies (Arnold et al., 2014; Bichsel, 2012; Norris & Baer, 2013), ethical issues surrounding data (Klein et al., 2019b). For a trusted LA, there is a need for a high level of data accuracy and effective intervention and visualization output to increase the trust of data and feedback as well as consistency between user needs and LA services provided.

To measure the level of dis(trust) of teaching staff in LA, there is a need to discuss the factors that have a significant impact on the trustworthiness of LA stakeholders and tools.

#### Trust factors in learning analytics

Previous research shows that trust issues are important factors that impact LA adoption. The work of ) suggests three main trust issues in LA: (1) numbers are subjective, (2) fear of power diminution, and (3) design & implementation. Their work investigates the perceptions of students and teaching staff about trust in LA. Furthermore, Klein et al. (2019a) proposed factors that affect trust in LA, such as privacy, alignment of technology, transparency and consent, ethics, beliefs and behaviors of faculty and advisors, organisational readiness, and capacity.

Other researchers considered trust to be the gateway to LA adoption, including power relationships, data ownership, anonymity and data security, privacy and data identity, and transparency (Drachsler & Greller, 2016). Individual online privacy is proposed to be an important factor in student trust in LA (Slade et al., 2019). Decision-making affected by decentralisation, lack of policies, trust in institutional commitment, and leadership have also been proposed as predictors of faculty and advisor trust in HEI in LA implementation (Klein et al., 2019a). A study by (Ciordas-Hertel et al., 2019) concluded that privacy is an important factor that affects trust in the LA infrastructure.

Researchers have identified various factors that are associated with the trust of LA users. Among these factors, privacy has been widely cited as a factor affecting trust in the LA adoption process (Drachsler & Greller, 2016; Klein et al., 2019a; Tsai et al., 2021b), followed by transparency and consent (Drachsler & Greller, 2016; Klein et al., 2019a; Tsai et al., 2021b). Therefore, it remains unclear which trust factors have the most substantial relationships with the trust of teaching staff in LA stakeholders and tools. Once the factors are known, institutional leaders or third parties (e.g., LA service providers) who want to develop strategies to create or maintain trust in LA are more likely to succeed in LA adoption.

#### Ethics and privacy

Privacy has been identified as the most critical factor related to trust in LA (Scheffel et al., 2014). Mutimukwe et al. (2022) considered trusting beliefs to be the extent to which higher education institutions are reliable in protecting personal information about users (e.g., students). The results of the Mutimukwe et al. study (2022) show that a high awareness of privacy risks and/or a low awareness of privacy controls among students can raise concerns about students’ privacy and possibly lead to students’ reluctance to disclose personal information. This may cause students to distrust HEIs. The study by Drachsler and Greller (2012) shows that two-thirds of the experts surveyed believed that LA would affect privacy and personal affairs. Thus, privacy should not be treated as a burden but as a vital ingredient to establish trusting relationships with stakeholders (Drachsler & Greller, 2016; Gašević et al., 2016a, 2016b; Gašević et al., 2016a, 2016b).

#### Transparency and consent

Trust and transparency are positively correlated(Rawlins, 2008), and are essential to build and restore relationships (Bandsuch et al., 2008). Organisations that facilitate and encourage public participation are more likely to be trusted and can empower participants to make informed decisions (Rawlins, 2008), which means that increasing transparency and accessibility in communication can improve trust. Therefore, a lack of transparency around personal data can exacerbate concerns about misuse of personal data, increasing the concerns of data subjects (Drachsler & Greller, 2016). Klein et al. (2019a) stress the value of including stakeholders and being transparent as critical factors in building trust. Thus, data storage, access, and manipulation should be done transparently and easily explainable with the consent of the interested parties (e.g., teaching staff and students).

Building trust in LA stakeholders and tools is essential to implement LA tools. Once trust is lost, it may have severe consequences for all parties involved. In cases when teaching staff lose trust in HEIs can particularly be harmful with detrimental social and ethical implications for the adoption of LA. Thus, it is necessary to explore the area of trust in LA stakeholders and tools and the factors that impact such trust.

## Methods

### Data collection

The current study adopted the instruments within the SHEILA framework^1^ to examine the extent to which teaching staff trust other relevant stakeholders and LA tools by administering a survey and to identify the factors that shape trust in LA by conducting interviews. Mixed methods were used to provide a more complete picture of the phenomena under study (Creswell & Poth, 2016). The study was conducted with the ethics approval from Monash university Ethics Committee under number 27304.

The study was conducted within HEIs in Saudi Arabia, where the data collection was initiated with the support of the co-author, Naif Aljohani, who distributed the survey through mailing lists and approached teaching staff at three Saudi HEIs. The survey was also distributed through social media, known to be used by teaching staff in Saudi HEIs. Data collected from Saudi HEIs lasted from early April to late August 2021. A brief introduction of LA was given to the teaching staff at the beginning of the interview and in the Qualtrics form.^2^

### Participants

#### Survey

The total number of responses was 103, of which 65 were complete (63.10%). There was almost the same number of responses from males and females—32 (49.32%) and 33 (50.77%), respectively. The response represented 20 Saudi HEIs from different disciplines. The number of years of experience of teaching staff ranged from one to 20 years. The majority of the sample were lecturers (n = 36, 55.38%), followed by teaching assistants (n = 12, 18.46%), assistant professors (n = 9, 13.85%), and then associate professors (n = 8, 12.31%). Almost the same number of teaching staff (50%) taught a subject of information technology (n = 20, 30.77%) and medicine and health science (n = 19, 29.23%), followed by 40% (n = 26) subjects within other disciplines (e.g. communication and media, social sciences). The participants had different administrative roles (e.g., deputy/assistant director (n = 8, 27.59%), head of the college (n = 6, 20.69%), Dean (n = 1, 3.45%), and others (n = 14, 48.28%).

#### Interview

A total of 24 individual interviews were conducted with teaching staff from three Saudi HEIs from different disciplines (for more information about the teaching staff sample, see Table 1). For the individual interviews with teaching staff, a snowball sampling technique was used to ask participants to guide the researchers to other possible participants (Robinson, 2014). The author, Naif Aljohani helped the first author approach a number of potential participants in one of the HEIs involved, as well as one participant in each of the other two HEIs, who in turn, helped in recruiting additional participants. In terms of the number of interview participants, Marshall et al.(2013) examined 83 qualitative studies of the information system in leading journals in information systems with respect to the number of qualitative interviews and suggested that the number of interview participants should be between 20 and 30. Saunders and Townsend (2016) argue that the number of participants depends on the quality of the responses to gather sufficient information on the research goal. Based on recommendations from previous studies on the number of participants, the number of teaching staff in this study was 24 from different disciplines in three Saudi HEIs.

**Table 1:** Participants’ information (qualitative data)

Pseudonyms	Gender	Discipline	Positions	University size
TS1	Male	IT	Asst. Prof	Large1
TS2	Female	IT	Asst. Prof	Large1
TS3	Male	IT	Asst. Prof	Large1
TS4	Female	IT	Asst. Prof	Large1
TS5	Female	Nursing	Asst. Prof/Admin	Large1
TS6	Female	Management	lecturers	Large1
TS7	Female	Accounting	lecturers	Large1
TS8	Female	Statistics	lecturers	Large1
TS9	Male	Educational technology	Asst. Prof/Admin	Large2
TS10	Male	IT	Asst. Prof	Large2
TS11	Female	IT	lecturers	Large2
TS12	Female	IT	lecturers	Large2
TS13	Female	IT	TA	Large2
TS14	Female	Early childhood	lecturers	Large2
TS15	Female	English	lecturers	Large2
TS16	Male	Medicine	lecturers	Large2
TS17	Male	IT	Asst. Prof/Admin	Medium
TS18	Male	IT	Asst. Prof	Medium
TS19	Male	IT	Asst. Prof	Medium
TS20	Female	IT	Asst. Prof	Medium
TS21	Female	Art and design	Asst. Prof	Medium
TS22	Male	Special education	Asst. Prof	Medium
TS23	Male	Engineering	Assoc. Prof	Medium
TS24	Male	Health services	Asst. Prof	Medium

*IT* Information technology, *Asst. Prof* Assistant professor, *Assoc. Prof* Associate professor, *TA* Teaching assistant, *Admin* administrator

The total number of teaching staff involved in the interviews included eight from a medium-sized HEI with a student population ranging between 10,000 and 30,000 and 16 teaching staff from two large HEIs with more than 30,000 students. University ‘Large 1’ and ‘Large 2’ differ in location and ranking, with ‘Large 1’ university being located in the west and ‘Large 2’ university being located in the south, as shown in Table 1. Participants were sampled from 13 colleges in the HEIs, and the selection process focused on diversifying disciplines; however, almost 50% were from the Faculty of Information Technology (n = 12), and 50% were from other faculties. A total of 24 teaching staff: 11 men (45.83%) and 13 women (54.16%), participated in individual interviews. The positions of the sample were as follows: (n = 14,58.33%) members of the staff were assistant professors (Asst. Prof), (n = 8, 33.33%) were lecturers, (n = 1, 4.16%) an associate professor (Assoc. Prof), and (n = 1, 4.16%) was a teaching assistant (TS). Three participants had administrative roles (Admin) (e.g., dean, vice dean, and academic director).

### Data collection procedure

#### Survey

The items used in the teaching staff survey were compiled from the questionnaire validated in the development of the SHEILA framework (Tsai et al., 2021a), including 15 expectation items and demographic and general questions about LA usage (for more information, see Appendix A2). The questionnaire measured the user’s expectations about LA by comparing ideal expectations (what users desire) and predicted expectations (what users expect to happen in reality). Predicted expectations ratings and the difference between predicted and ideal expectations were considered as trust indicators as per the work presented.in (Tsai et al. 2021b). According to this approach, low predicted expectations indicate that respondents were less confident in LA tools or the ability of their HEIs to implement or adopt the statement in reality. Furthermore, the wider the gap between ideal and predicted expectations (with the latter being lower), the greater the distance between the desired state and the belief that it would be achieved in reality. The expectation items were anchored on two seven-point Likert scales, ranging from 1—strongly disagree to 7—strongly agree. The survey was translated into Arabic by a professional translator. Two PhD students for whom Saudi Arabic is the first language checked whether the questions were clear after the translation. Some changes were applied to the survey, including more common words in the Arabic culture, such as ‘educational data analysis’ as a replacement for ‘learning analytics’.

#### Interview

Before conducting the interviews, emails were sent to the participants along with an explanatory statement and interview questions (see Appendix A1). The interview was conducted after receiving the consent of the participants in advance to respect the privacy and autonomy of each person. During the free time of the participants, prearranged interviews with teaching staff from three Saudi HEIs were conducted online through Zoom. All interviews were recorded for audio and took between 30 and 45 min.

### Data analysis

#### Survey

To answer RQ1, *To what extent do teaching staff trust LA stakeholders and LA tools?* we conducted an exploratory analysis of the survey using descriptive statistics, including mean and standard deviation. Paired t-tests were also performed to compare responses between ideal and predicted expectations (teaching staff survey protocols shown in Appendix A 2). For more information on the results of the t-tests, see Appendix B 1 and B 2.

#### Interview

To answer RQ 2, *What factors shape teaching staff trust in LA stakeholders and tools?* we used the interviews that were recorded and transcribed verbatim to capture the full scope of the participants’ narrative. The semi-structured interviews (as shown in Appendix A1) were then analysed using Clarke and Braun’s well-established Thematic Analysis (TA) guidelines(Clarke & Braun, 2017). Data were analysed using NVivo^3^ and MAXQDA^4^ as computer-assisted qualitative data analysis software systems. Given that most of the interviews were conducted in Arabic and only three were conducted in English, MAXQDA was a suitable choice, as it supports both languages. However, NVivo was used to calculate inter-rater reliability using English transcripts, as it was the tool of choice for the two coders.

All data was analysed to identify certain patterns and classified and organised accordingly in the initial themes according to the SHEILA coding scheme (the reference should be Tsai 2021a for the article Connecting the dots: An exploratory study on learning analytics adoption factors, experience, and priorities). However, both deductive and inductive methods were applied, where the deductive method was applied using predetermined codes (i.e., SHEILA coding scheme), while the inductive method was applied to identify emerging new codes. To ensure coding consistency and resolve the disagreement, two coders carried out the inter-rater reliability test. The first coder (the first author) explained the initial coding scheme to the second coder. Then, two coders were involved independently in four rounds of co-coding with two different English transcripts, as this was a common language between them. Two meetings were held for each transcript to discuss the coding process, such as the code name, code definitions, and emerging or splitting codes. Based on the coding comparison query of two interviews, the inter-rater reliability of coding indicated a good level of agreement between the coders (Cohen’s Kappa = 0.65) as indicated by (McHugh, 2012). After that, the first author coded the rest of the interview transcripts.

The final coding scheme ended up with four main themes, incorporating both the initial SHEILA coding scheme and the newly developed themes and sub-themes from the collected data. The themes included *concerns*—related to LA functionality, data, ethics, or privacy-related issue, *teaching practise*—challenges, intervention, and curriculum design-related activities, *educational data and communications*—topics related to data types, data analysis center, training, and communication types, and *teaching staff perceptions*—desirable features of LA or data types, experience with LA, and trust and distrust in LA tools or LA stakeholders. The *concerns* theme was derived from the SHEILA coding scheme, *teaching practice* and *educational data and communications* are two new themes used to organise predefined SHEILA themes, while *teaching staff perceptions* was a new theme.

Each of these themes contained three to six sub-themes (18 in total), and the total number of codes under these sub-themes was 42 codes (for more information regarding thematic codes, including details about predefined and new themes and sub-themes, see https://bit.ly/Themes__Codes). The majority of the quotations were originally collected in Arabic and were first translated into English before being included in this paper. Quotes from staff interviews included in this paper were labelled with TS (teaching staff), while the numbers refer to identifiers for the teaching staff involved. It is important to note that the selected quotes represent the original responses, with a few minor edits, e.g., omitting phrases such as’like’,’that’, or’I mean’ because they were not considered significant for the study.

## Results

### To what extent do teaching staff trust other relevant stakeholders and LA tools? (RQ1)

**Fig. 1:**
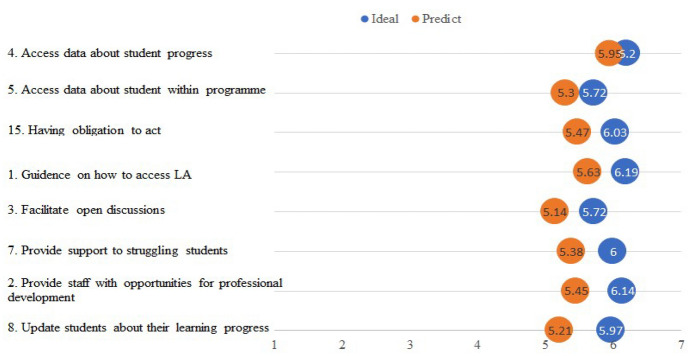
Summary of the teaching staff’s trust in LA stakeholders, showing the ideal and predicted expectations

**Fig. 2:**
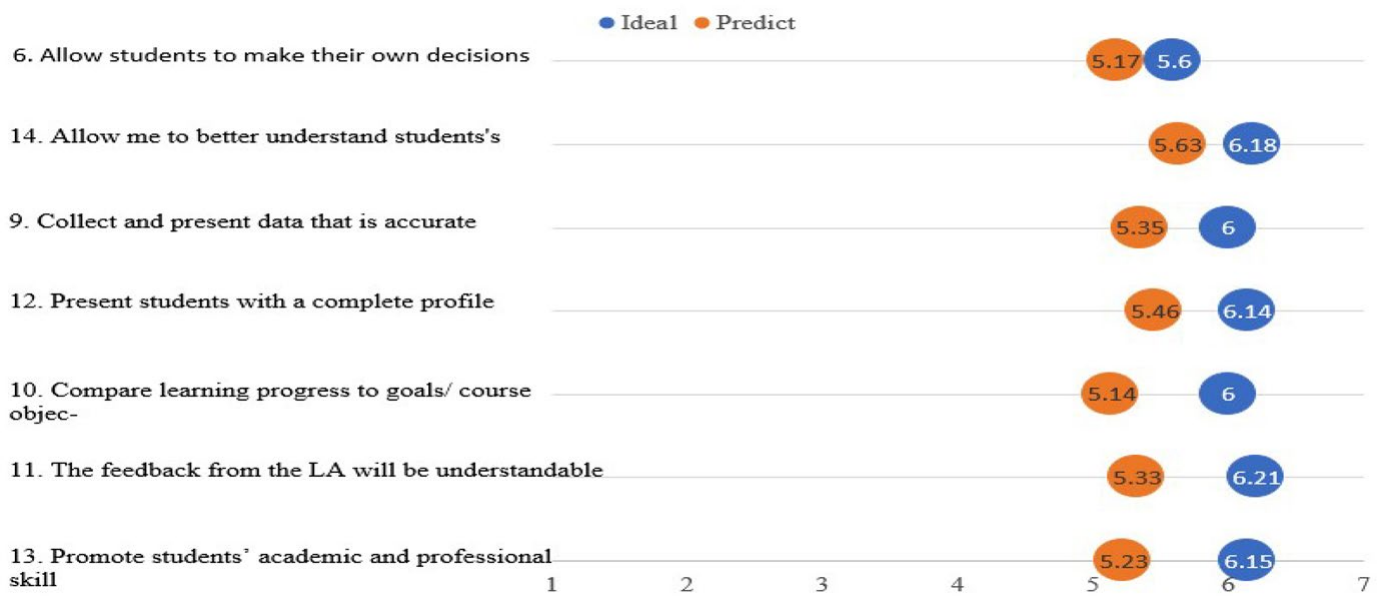
Summary of the teaching staff’s trust in LA tools, showing the ideal and predicted expectations

Figure 1 and Fig. 2 present the results of the teaching staff survey (ideal and predicted expectations). Figure 1 presents the items related to trust in stakeholders (e.g., HEI), and Fig. 2 presents the items related to trust in LA tools. They are also elaborated on below by focusing on the items that had the highest and lowest differences between the ideal and predicted expectations.

In terms of the expectations of the teaching staff in their HEIs, the mean differences between the ideal and predicted expectations ranged between 0.25 and 0.76, as shown in Fig. 1 and in more details see Appendix B 1. When it comes to expectations management, the small gaps between expectations and ideal expectations point to areas where less attention or effort is needed. For example, the confidence level among respondents with regard to Item 4 (Access data about my students’ progress) was relatively high, as the absolute difference between ideal and predicted expectations was the lowest. Item 4 also had the highest average rating of the ideal expectations, indicating that the participants found this aspect to be the most desirable to happen in reality. A paired t-test did not show significant differences between the two expectations, with a small effect size for both items (t = 1.814, p = 0.074, Cohen’s d = 0.266). On the other hand, item 8 (Update students about their learning progress) had the largest rating gap between predicted and ideal expectations. This indicates an aspect of the highest distrust among respondents. The paired t-test showed a significant difference between the two expectation types with a medium effect size (t = 3.301, p = 0.002, Cohen’s d = 0.55).

In terms of the expectations of the LA tool, the mean differences between the ideal and predicted expectations ranged between 0.43 and 0.92, as shown in Fig. 2 and in more details see Appendix B 2. Item 6 (Allow students to make their own decisions) had the lowest average rating of the gap between predicted and ideal expectations; however, it had the least ideal expectations compared to other items. This indicates a less desirable but high level of trust in LA tools. The paired t-test of item 6 showed a significant difference between the two types of expectations with a small to medium effect size for item 6 (t = 2.301, p = 0.025, Cohen’s d = 0.32). On the contrary, item 13 (Promoting student academic and professional skill development) had the largest rating gap between predicted and ideal expectations with a large effect size (t = 4.490, p = 0.000, Cohen’s d = 0.7). This indicates an aspect of the highest distrust among respondents of LA tools in terms of providing professional skill development.

### What factors shape teaching staff trust in LA stakeholders and tools? (RQ2)

During the interviews, the participants discussed the factors that impact their trust in the LA stakeholders (subsection 4.2.1) and the LA tools (subsection 4.2.2).

#### Dis(trust) in LA stakeholders

Much of the discussion about dis(trust) in LA stakeholders focused on stakeholders’ (e.g., HEIs and third party) competence to adopt LA or handle issues related to ethics and privacy.

##### Competence

*HEIs:* trust in competence is based on experience and expertise (Terwel et al., 2009). It can also be evaluated as the soundness of HEIs to have technology capabilities (Mcknight et al., 2002). Four participants from large HEIs shared their perspectives related to the issue of trust in stakeholders to adopt LA based on current competence and expertise in e-learning systems; they believed that their HEIs had a strong e-learning infrastructure that would allow them to adopt LA effectively. In a similar vein, TS8 commented on the capabilities of teaching staff in specialised departments, such as statistics, to handle data analysis and become internal and external experts. TS8 explained this point stating, “We have educated staff who can perform the task of analysing data in the Statistics Department. We do not need an external party to use the data. We have the competence to provide consulting to external parties.” Another point of view of TS4, who worked at a large HEI, stated that the current competence of the HEI (large HEI) allowed the HEI to be a third party for other HEIs due to their experience with relevant data analysis. TS4 said.

“It depends on the size of the university and the expertise they have; there are new universities that may benefit from the experiences of other universities, the big universities like [university names]. These all have research centres, and they analyse data. I think they don’t need to deal with a third party; maybe they can be a third party for a young university. I prefer, that the big university will be the third party of a young university, and they will send a consulting to it, and with training, and in the end, they will be able to establish LA for themselves.”

*Third-party:* regarding the dis(trust) in third-party competence, the key issues were raised: a) the key business goal of a third-party and b) prior experience with different e-learning platforms. For example, TS1 explained that trust in third parties derived from their experience in LA as their own business. TS1 explained these points by saying that third parties have the capacity and expertise and can contribute very well to the data." Most of the teaching staff use the Blackboard Learning Management System (LMS) for their daily tasks. A positive experience with another educational technology, such as Blackboard, would have an impact on the trust of the LA third party, as reported by TS2, "They specialise in this field. We use a lot of software, and they are innovating in this field. Even if it were for data analysis, it would be better if they were specialised companies." One teaching staff shared her thoughts and other teaching staff’s thoughts toward having a contract with a third party, partly due to unfairness and partly due to the lack of awareness about the existing situation (e.g., current data practice, teaching needs, policy and procedure). For example, TS7 stated that.

“The [university] has suggested that teaching staff be evaluated based on instruments administered by external software systems or external companies. And many teaching staff have opposed it. [...] But many argued that an external evaluation would be unfair. Data analysis at the university is better. Many external factors affect the information we provide and its nature. The university wanted an external party not to be biased. But I expect that if an external party analyses the data, whether about students or teaching staff, they will not be fully aware of our situation. An internal party would be better.”

##### Privacy and ethics

*HEIs:* participants highlighted two privacy and ethical issues that could impact their trust in HEIs, which are access rights, transparency and consent.

- *Access rights:* as more students and teaching staff communicate on different e-learning systems, concerns have grown about who can access the data and with what access rights. This concern may affect the trust of teaching staff in the HEI in terms of handling privacy and ethical issues. Four teaching staff in medium and large HEIs had concerns that the absence of data governance in terms of accessing data would violate teaching staff data privacy and misuse of the data. For example, TS10 stated that,
  “Everyone should have limits in accessing the data. Unfortunately, this is not the case. Even internally, some universities do not have regulations. It is possible that they access [data], work on the data and analyse them. It is true that they are all from within the organisation, but there is no governance on this matter. This is a matter on which all government institutions must work on. seriously.”

b) *Transparency and consent:* they were viewed by teaching staff as mandatory in the data collection process. However, the process of consent is not clear to teaching staff, which may affect their trust in their HEIs. This indicates that consent is not consistently introduced to all teaching staff. The reasons might be related to the absence of a clear policy on the data collection process. As TS15 noted, “There is no clear policy about storing or collecting data; private data that reveal the identity of students are not used. But there is no policy on the type of data that need permission and the type of data that do not need.” TS24 was also concerned about sharing data without being adequately informed and aware of the nature of the data or to what extent the data can be used and states that “The university always ask us to update our data, but they do not give us the option to opt-out. Everything collected from us needs to be with consent.” However, another perspective related to the level of transparency in the collection of data by HEIs was voiced by TS21, “On the Blackboard [LMS] at the top of the screen, when students submit the assignment, they can see that the university has the right to use or share the data.” Furthermore, TS3 had high trust in HEIs to allow teaching staff to exercise their right to opt-out, especially special data. TS3 states that “Yes, for special kinds of data and for a special purpose, of course, I think the university can allow this.”
  *Third-party:* In terms of trust in third parties, two concerns were identified in teaching staff interviews, including data sharing and ownership.

- *Data sharing:* there were divergent opinions toward outsourcing or sharing with external parties who can access and process educational data on behalf of the HEI. For example, TS6 expressed his concerns about outsourcing data and emphasised that obtaining the user’s consent is required before outsourcing data processing. TS6 stated that he “wouldn’t have any concerns if that is exclusively written in a consent that the data would be shared with a third party. […] I mentioned that there should not be that much personal information that you know could be harmed. If somebody knew about it.” TS17 raised concerns that LA is used by advertising companies rather than providing educational services, “If, for example, without names, without identification numbers, general data, there are no problems, but the fear for those who use student’s data with names, mobile numbers and emails, it is possible that these are sold to advertising companies.” TS5 pointed out that concerns related to outsourcing data depend on the types of data that have been shared and said that
  “The [university name] has contracts with many companies, including Coursera. We do not have a problem as long as they are not private data. For example, my bank account and the civil registry number are my private data. I do not allow the university to share personally identifiable information with a third-party. But if there is anything about teaching and learning, I have no problem.”
  The last aspect that has not been previously reported in the literature was the concern about *sharing the data* with an overseas third party. In the interview, TS11 talked about her concern about “sending educational data out of Saudi Arabia” as follows:
  “It is sensitive information. This analysis can contain data that predict the nature of student thinking. This information is critical to the university, so third-party companies should not be located outside the country. This is very important. I mean, even these companies must be authentic inside the country.”
  *b) Ownership:* it is another aspect that caused participants to hesitate to have services or trust a third party in terms of handling learning analysis tasks. According to TS4, she had no concerns about having an external consultation. However, TS4 was concerned about “the possibility of having the data owned by a third party who may take control over or mishandle teaching staff data”, and she also noted
  “We can ask for a consultant who gives us a consultation, we do a design, we meet him, and we ask him what he thinks, but don’t let everyone settle the system, and then he owns the data and becomes responsible for it. "

#### Dis(trust) LA tools

The current adaption level of LA in Saudi HEIs is in an early stage; therefore, participants discussed their dis(trust) issue in LA based on their expectations of LA usefulness, concerns related to human contact with the use of LA, data accuracy, and prediction precision.

##### Usefulness

- *Institutional level.* All teaching staff viewed LA as having a largely positive impact on their campuses. For example, most of the teaching staff believed that LA could create benefits that would inform learning design and teaching, better productivity, lead to a new source of funding for new educational software systems in HEIs, and increase the competitive value of HEIs. For example, TS2 stated that
  “it can also improve the university’s image and reputation to increase its ranking. It can enhance the image and the university’s reputation to attract more students, especially universities, when they create their programmes to be for a fee. Analysis is definitely important.”
  Furthermore, TS9 added that there would be an increase in online programmes for some theoretical disciplines, which can increase the need for LA. He also had a positive view of the usefulness of LA in providing data to the targeted audience with less time and effort.
  “Since the beginning of the pandemic, the universities have been displaying weekly data related to e-learning use as a video or infographic. So instead of collecting data and creating an infographic and converting it to PDF, then sending it [to the ministry, university administration]. We linked the dashboard to a database and the data is automatically updated. This can save team effort. We design only one page [i.e., dashboard]. The project also had a good resonance at the ministry level.”

b) *Teaching and students’ level*. The acknowledgement of culture may enable the full benefits of LA to be realised. A respondent, who experienced different cultures (Western and Saudi cultures), shared her perspectives with regard to the relationship between teaching staff and students and expected that LA would provide a complete picture of students’ needs without making students feel embarrassed to share their weaknesses or needs. TS20 noticed that
  “Saudi students may not be frank with all the answers because it may show their weakness in front of the teacher. I expect the analysis of educational data will support students or save a lot of effort between students and teaching staff because teaching staff can learn about different needs of students.”
  In addition, all teaching staff had reasonable expectations and a high confidence in the usefulness of the LA to improve the materials provided to students according to their level of knowledge, as also aimed in some early LA systems(Ali et al., 2012), starting from basic to advance, doing their work with students more effective and efficient. As one of the teaching staff stated,
  “We can know the weaknesses of the students and support them. We can amend the content of the lecture curriculum. We can provide more basic information for those who had a weakness, and we can add more advanced information for those students who were more advanced."
  Although the teaching staff had a high level of trust in the usefulness of LA, they were concerned about human contact, data accuracy, and prediction precision.

##### Human contact

The effects of LA are not all positive; however, while LA can improve students’ experience or teaching practice, teaching staff prefer meeting students and discussing their needs more than depending on technology for communication. Two of the teaching staff argued that improving the “human–human” relationship would not be possible to replace it with “human-data-human” interactions. TS5 stated that.

“What is between a student and me is a transparent relationship, which means that if the student has a problem. He comes and says to me, Doctor, I have this problem. This is what I am trying to do. At the beginning of the course, I can ask the students to come back to me, or this is a kind of relationship that we call a trust relationship based on transparency between the student and me. The data are useful in developing teaching methods, evaluation, and conditions, of course, and student and all things followed, but the human relationship. I don’t think the data will even provide benefit from this aspect.”

##### Data Accuracy

Six codes were developed to capture concerns about data accuracy, including *outdated data, different data channels, lack of data governance, power relationship, response bias* and *technical issues*.

- *Outdated data,* briefly defined, are the data that are out of date or the data that should be replaced by new data. TS3 expressed his concern that collecting and using the students’ data during different temporary issues that the students may face at some point in their studies (e.g., financial, family, or health) may cause LA outputs that do not reflect the student’s current or future situations. For example, TS3 reported that

“Sometimes, for students, under some special circumstances, for the time being, the data and responses [to surveys] are reflective of special circumstances. Therefore, if we use the [old] data, it might not be relevant under the current circumstances of the students. When we analyse these data which are not relevant, we might get a wrong result. So, whenever we go for this kind of analytics, we should have random data and real at the different stages and not some kind of data recorded under special circumstances.”

b) *Different data channels* were perceived as especially significant for accurate analytic results. Interviews with the participants commonly stressed difficulties in collecting data that teaching staff needed for their practice. TS17 discussed this point as

“Videos are important to me. For example, if I ask students, especially in programming and computing subjects, to watch a video [obtained from another source, such as YouTube] about a programming language, the data collected can sometimes be deceptive because I can give them a video name. So, they go to watch the video on Blackboard [LMS], or they can watch it on YouTube or search for it on any other video platform. This kind of data is difficult to track and to make a judgment on [e.g. analysis].”

c) *Lack of data governance.* The lack of data governance is another concern that is associated with the accuracy of data. It refers to “the exercise of authority and control over the management of data assets” (Allen & Cervo, 2015, p. 11). Three teaching staff considered the absence of data governance to be a concern that leads to distrust in data-derived insights due to inconsistencies in data collection, modification, and integrity. For example, TS10 stated, “Yes, and it is one of the problems. If we go back, let us say that the roots of these problems are governance. When we do not know who is modifying the data and who is accessing the data.”
d) *Power relationship* refers to the ability of an HEI to exert control or direct students. Although not mentioned by many teaching staff, four teaching staff shared their concerns about data accuracy issues due to the lack of credibility in collecting data, especially those collected via surveys. This is because students have to complete a survey to view their overall final grade, which increases the possibility of randomness in students’ answers. The participants also expressed concern that collecting data through surveys is the only way to get students’ feedback on subjects or teaching staff feedback; however, completing the survey by giving random responses by students would not make the survey a suitable tool for data collection and later as a useful data source for LA tools, as observed by TS17,

“Unfortunately, sometimes the university forces the student to complete the survey to allow them to see their final grade, so students give random answers; the students’ responses may be all ’agree’, which diminishes the credibility of the survey.”

*e) Response bias* is another concern that refers to the factors that influence the way responses are given. TS12 pointed out how the natural relationship between students and teaching staff impacts how the response is given, which makes teaching staff prefer a survey with free text responses to the questions in which students can describe their challenges or thoughts. TS12 elaborated on this point as follows

“Not all students responded to the survey. There will be a degree of randomness; as we say, it is not taken by 100% [of students], but it can give you some insights. In the university, in every academic course, the student does not get or does not receive the grades of the course until she assesses the professor’s course, and we do not depend on this survey. I mean, you notice that if the teacher is excellent or let us say a little strict in terms of grades, you will find that her assessment is declining, unlike the teacher who is a little permissive, and her assessment is high”.

(*f) Technical problems* may cause inaccurate data in specific contexts).

Two teaching staff noted a technical issue in the e-learning system setting. Consequently, the system did not work as expected by the teaching staff, and, therefore, incorrect data were recorded, which may, in turn, impact the reliability of LA results. As such, inaccurate attendance records force teaching staff to record attendance manually. TS17 discussed this point as follows.

“The problem is the credibility of the existing data. I mean, I’ll give you an example. When I put in the Blackboard [LMS] settings, “if the student attendance percentage is below 50%, then the Blackboard should record the student as absent”. However, I see his name in front of me [the student was attending the class online], but the Blackboard setting recorded that students attended less than 50.

##### Precision of prediction

Two teaching staff members also acknowledged the precision of prediction issues due to *the inconsistency in the student data*. TS7 made a point that student performance during the same semester would be changeable, which means that student performance at the beginning of the semester is not the same as at the end of the semester. In other words, the high performance of the students over a period of time would not be sufficient to build a judgment on students’ overall performance. TS7 also suspected that the results of the analysis of students’ data from a specific cohort could not be used as a reliable source (e.g., to predict the risk of failure) for another cohort due to their different educational levels and data dynamics. Limited generalisability in predictive modelling has also been observed in the LA literature (Gašević et al., 2016a, 2016b). TS7 stated that,

“When we collect data from students in one semester, we will have students with different levels in another semester. So, I expect that the student’s data will not affect much. You will not be able to generalize this to everyone. But let us talk in general about academic data [e.g., course curriculum]; the analyses can help us because they are stable data that change after five years. But the levels of the students change continuously because the curricula can change and therefore the level of students entering the university is variable.”

The second significant concern related to the precision of predictive analytics was the absence of psychological factors involved. A view shared by two participants who had concerns with the type of data used to predict students’ performance or identify at-risk students, as it could inaccurately represent students’ performance. For example, TS17 stated that.

“Here, we have an additional problem. What are the weak skills of the student? Is it the grade of the students? Was the student tired when attending the exam, did not remember well, or had family circumstances? Is the student not interested or careless? Does it mean that the student’s weakness is that he is introverted and does not participate? I mean, a student who does not participate but responds with high grades? This is his capability. Students are of different characteristics. This is difficult to analyse. It may be necessary to include psychological factors or collect data for a personal study. This is important. Electronic personality tests."

This final point is also echoed in the recent studies in LA that emphasised the critical role of data about individual differences in predictive modelling (Jovanović et al., 2021).

## Discussion

### Summary of findings

Several significant findings emerged from the analysis. First, the teaching staff survey indicated that teaching staff had a high trusting expectation toward LA stakeholders in the area of “access” (items 4,5). They also had high trust in LA tools in the areas of “allowing students to make decisions” (item 6) and “understanding student performance” (item 14). Interviews with teaching staff indicated a high level of trust in LA tools in terms of their usefulness and a high level of trust in HEIs and third-party competence. Table 2 Summary of teaching staff dis(trust) in LA stakeholders and tools. The factors of distrust (trust) are highlighted in the survey (S) or the interview (I).Table 2 lists all trust and distrust factors in both LA stakeholders (e.g., HEIs, third-party) and LA tools. They are also elaborated on below.

**Table 2:** Summary of teaching staff dis(trust) in LA stakeholders and tools. The factors of distrust (trust) are highlighted in the survey (S) or the interview (I)

Dimensions	Trust	Distrust
HEIS	Competence(I)	Privacy and ethics(I) (e.g., access right, transparency and consent)
	Providing access(S)	Providing professional development(S) Update students about their learning progress(S)
Third-party	Competence(I)	Competence(I)
	Privacy and ethics (I) (e.g., sharing educational data)	Privacy and ethics (I) (e.g., sharing personal data, ownership)
LA tools	Usefulness(I) (e.g., institutional,	Data accuracy(I)
	teaching and learning level)	Understandable(S) Human contact(I) Precision of prediction(I) Usefulness(S) (e.g., Promote students’ academic and professional skill development.)

Teaching staff dis(trust) in HEIs: Different studies discussed factors that impact trust in HEIs in different ways, such as the lack of consensus on staff capabilities or the prospect of losing professional autonomy (). The findings of the current study confirmed the factors mentioned in (Alsheikh, 2019), showing that HEI competence is a significant factor to be considered in LA adoption and in estimating teaching staff trust in HEI. Moreover, the current study offers new insights into the literature by indicating that teaching staff’s trust in HEIs competence may relate to their experience with the current HEIs’ technology infrastructure or the experience and capacity of HEIs in data analysis (e.g., dedicated centres or other organisational units). In terms of HEI services, the survey of teaching staff revealed a level of distrust in HEIs to provide professional development opportunities. This suggests that teaching staff should have organisational support and learning opportunities to learn a set of skills to use LA tools, extract valuable information from educational data, and determine which type of data analysed is more relevant to the HEI goals (Tulasi, 2013).

The results of the current study in connection to privacy and ethics are in line with previous research (Slade et al., 2019) showing that stakeholders (e.g., teaching staff) had a high level of trust in HEIs to handle privacy and ethical issues; however, the current study yields an interesting addition, which is a concern related to the absence of data governance in terms of access rights and how this could lead to data privacy violation. Such concerns warrant the need to establish data governance guidelines, as suggested by (Elouazizi, 2014). Furthermore, the results of the survey of teaching staff in the current study and those presented by ) revealed a high level of trust in HEIs in allowing access to students’ data within the degree programme. This indicates a percentage of satisfaction among teaching staff with the’access’ service provided by the HEI. To sum up, our attention to the literature and the findings of the current study was drawn that privacy and ethics have so far been regarded as difficult issues that need to be addressed properly.

Teaching staff trust in third-party: Our findings are consistent with previous work (Tsai, Whitelock-Wainwright, et al., 2021b) showing that stakeholders (e.g., students, and teaching staff) were concerned about their data being sold to advertising companies that can take advantage of the data. This result caused stakeholders to be concerned about commercial targeting. In many cases, sharing data about teaching staff and students with third parties for unintended purposes should not be a common practice, and institutions should be aware of and transparent about data-sharing processes. Another related point to be considered is the type of data that is shared; in which there was a consensus among teaching staff that private data should not be shared with a third party, but they do not mind sharing their educational data. Data ownership and exchange are the two main challenges when working with a third party (Leitner et al., 2018). These two challenges align with the findings of the current study, in which the teaching staff were concerned about data ownership and what types of data should or should not share with third parties.

Furthermore, teaching staff expressed trust in third parties based on their previous experience with another IT third party (e.g., Blackboard). Teaching staff believed that a third party would provide effective LA services and features when LA tools are their main business focus. However, some concerns among teaching staff were raised regarding the handling of LA services by third-parties, including concerns of unfairness due to the lack of third-party awareness of the current situation of HEIs. In general, gaining the trust of teaching staff should be part of the objectives the third parties should aim to achieve. This is because trust gives third parties a competitive advantage over their rivals. In general, if a third party sets high expectations but does not live up to them, the relationship will quickly decline, and both parties will be upset. In contrast, the relationship will be harmonious if expectations are met (Digital Promise, 2019).

Teaching staff trust in LA tools: different issues have been discussed in terms of teaching staff trust in LA tools, one of which is data accuracy. The current study confirmed that teaching staff had relatively low trust in data accuracy, as also reported in the literature (). However, the interviews in the current study expand on the reasons behind the lack of accuracy of data (e.g., outdated data, lack of data governance and differences in data channels). In terms of outdated data, the current study and the findings of the study by (Klein et al., 2019b) confirmed the problems related to inefficient data updating processes that may impact providing real-time data. This finding suggests a significant opportunity to increase trust in data by treating the data as an asset and effectively managing that asset regularly. Furthermore, the results of the teaching staff survey in the current study showed a low level of distrust in LA as a tool that is understandable, which has also been shown in (Tsai et al., 2021a, 2021b). It can be argued that unclear visualisation of data not only deters trust but can also result in a complete lack of use (Klein et al., 2019b).

There are essential differences in the findings of this study compared to the literature that are worth mentioning. The power relationship has been described differently in the literature. It was discussed as the asymmetry of power ingrained between higher education and students (Prinsloo & Slade, 2016; Slade & Prinsloo, 2013) or making students increasingly dependent on institutions to provide feedback rather than develop their metacognitive skills and dispositions(Buckingham et al., 2012), causing a sense of powerlessness and exploitation. Furthermore, teaching staff, as reported by (Tsai et al., 2021a, 2021b), considered the power relationship an issue they face in managing expectations from managers and students. The current study confirmed that the power relationship could also be considered as a concern related to data accuracy, where the HEIs asked students to complete the survey as a condition to see the final result, leading to random responses and inaccurate data recorded. Response bias is another concern that was observed among teaching staff, which is based on the relationship between students and teaching staff. This indicates that data collection through surveys should be improved, as data collected from surveys can be used as a data source for LA. Furthermore, teaching staff from different cultures can consider the usefulness of LA differently. For example, Tsai et al., (2021a, 2021b) reported that the teaching staff at an HEI in the UK believe that LA requires additional demands on time and effort. However, the findings of the current study show that LA would save time and effort and help teaching staff better understand their students, especially in a culture such as Saudi Arabia, in which students feel embarrassed to share their weaknesses. Furthermore, the current study also shows that the teaching staff had a high level of trust in the usefulness of LA as a tool to improve the educational experience in general and achieve the HEI objectives. This finding was confirmed by (Alsheikh, 2019), who stated that Saudi HEI staff and teaching staff recognised the importance of big data analytics to support decision-making.

### Implication for adoption

The current results have several implications, and we summarise those implications into several recommendations as follows.

#### Improve data accuracy

To develop and maintain confidence in learning analytics, institutions must monitor data quality, reliability, and validity to develop and maintain trust in learning analytics. One of which is improving the methods and selecting the time appropriate to collect the survey data (as one of the data sources of LA) to avoid random data that may occur due to the power of the relationship between HEIs and students in the way data are collected.

#### Set a policy for data sharing and ownership

Data used for learning analytic purposes must comply with institutional data policies. Third parties should also be subject to the same data protection rules that the institution follows, such as not being allowed to share or use data for unintended purposes. Thus, setting a privacy policy is necessary before conducting a contract with a third party, which can be done by following the new Saudi Personal Data Protection Law (PDPL), which will be enforced in March 2023 in the Saudi government and the private sector (SDAIA, 2022).

#### Enhance the consent-seeking process

It is necessary to obtain informed consent from the teaching staff. When seeking consent, HEIs should provide detailed information about the terms and conditions of the data collection and use it to elicit trust from teaching staff in the use of their data. Consent options must be defined explicitly and meaningfully, and the expected impact of consent grant or withdrawal must be clarified. This process is acknowledged as a requirement in Europe (GDPR, n.d.) and in the Saudi Personal Data Protection Law (PDPL) once implemented.

#### Establish data governance guidelines

Data should certainly be treated as a valuable asset with meaningful use by setting data governance. Considering the implementation of the data governance framework, teaching staff are likely to feel a greater sense of commitment towards the HEIs and take a part of the responsibility to govern the data and ensure its protection.

## Conclusion and limitations

The findings of this study suggest that trust should be conceptualised not only as a calculative orientation toward risk, usefulness, or data accuracy but also as a social orientation towards stakeholders, such as HEIs and third-party. The result shows that teaching staff have a high level of confidence in the ability of HEIs to adopt LA and in the usefulness of LA tools; however, they are less trustworthy in third parties to deal with the issues of privacy and ethics and in data accuracy. This study advances our understanding of LA trust and can help HEIs and organisations providing LA services (mostly referred to as third parties in the current paper) develop and maintain LA tools considering trust factors and, therefore, can gain trust from teaching staff. Given the importance of trust for the success of LA adoption, HEIs should prioritise the teaching staff’s trust in LA through actions to better serve the goals of teaching staff as primary stakeholders in LA.

This study has three main limitations that could be addressed in future research. First, the study involved only teaching staff. Second, this study focused on the trust of teaching staff in specific stakeholders (e.g., HEI and third parties). Third, this study examined only a few trust factors (e.g., competence, ethics and privacy, data accuracy, and LA usefulness). Fourth, this study had a sample size that is relatively small for a survey study. Therefore, future studies should investigate trust factors from the perspective of the student, institutional leaders, and other relevant stakeholders. Furthermore, future studies can include trust in the teaching staff themselves, between teaching staff and other teaching staff, or between teaching staff and students. Furthermore, more trust factors (e.g., leadership) need to be examined within different HEI types (e.g., small, medium, large). Lastly, future research should include a large size of survey sample across differ contexts to improve the study power and the generalizability of the findings.

## Appendix

A Appendices related to methodology

See Tables 3 and 4

**Table 3:** Teaching staff interviews questions

Themes	Questions
Purpose	1. Learning analytics benefits from a range of education data including academic data, personal data, and engagement data collected from online or physical learning environments. What do you think would be legitimate purposes for the university to use such data?
Teaching needs	2. What kinds of data would be particularly useful to you in improving students’ educational experience in a module/course/programme that you are responsible for?
Teaching needs	3. What kinds of data would be particularly useful to you in your professional development?
Teaching needs	4. Do you see any challenges in offering teaching and learning support to your students?
Teaching needs	5. Do you see any ways learning analytics could be used to address these challenges by taking advantage of student data or data about your teaching performance?
Ethics and privacy	6. Do you consider there to be any ethical or legal issues concerning the use of student data or data about your teaching activities and effectiveness?
Educational support	7. Here are some examples of ways the university could use learning analytics to enhance learning and teaching. Which of these uses of do you think would be useful (multiple choices)? Please pick one to share why it is useful or not useful after the poll a) To improve the relationships between students and teaching staff or tutors b) To improve the overall learning experience and well-being of students c) To identify a student’s weaknesses in learning and suggest ways to improve upon this d)To alert teaching staff early if students are at-risk of failing a module or if they could improve their learning e) Identify the optimum pathway for students to achieve their learning goals f) Present students with a complete profile of their learning in each and every module g) Present teaching staff or tutors with a complete learning profile of their students h) Present teaching staff or tutors with a profile of their teaching activities and effectiveness
Intervention	8. How do you think teaching staff and tutors should approach the analysis results of student data?
Concerns	9. Are there any concerns you would have in incorporating learning analytics into teaching?
Final remarks	10. Do you have any suggestions for the adoption of learning analytics at the University?

**Table 4:** Teaching staff questionnaire

1. The university will provide me with guidance on how to access educational data analysis about my students	(Tsai et al., 2021a)
2. The University will provide staff with opportunities for professional development in using educational data analysis for teaching	(Tsai et al., 2021a)
3. The university will facilitate open discussions to share experience of educational data analysis services	(Tsai et al., 2021a)
4. I will be able to access data about my students’ progress in a course that I am teaching/tutoring	(Tsai et al., 2021a)
5. I will be able to access data about any students within a programme	(Tsai et al., 2021a)
6. The educational data analysis service will allow students to make their own decisions based on the data they receive	(Tsai et al., 2021a)
7. The university will provide support (e.g., advice from personal tutors) as soon as possible if the analysis of a student’s educational data suggests they may be having some difficulty or problem (e.g., underperforming or at-risk of failing)	(Tsai et al., 2021a)
8. The university will regularly update students about their learning progress based on the analysis of their educational data	(Tsai et al., 2021a)
9.The educational data analysis service will collect and present data that is accurate (i.e., free from inaccuracies such as incorrect grades)	(Tsai et al., 2021a)
10. The educational data analysis service will show how a student’s learning progress compares to their learning goals/the course objectives	(Tsai et al., 2021a)
11. The feedback from the educational data analysis service will be presented in a format that is both understandable and easy to read	(Tsai et al., 2021a)
12. The educational data analysis service will present students with a complete profile of their learning across every course (e.g., number of accesses to online material, learning outcomes, and attendance)	(Tsai et al., 2021a)
13. The feedback from the educational data analysis service will be used to promote students’ academic and professional skill development (e.g., essay writing and referencing) for their future employability	(Tsai et al., 2021a)
14. The use of educational data analysis will allow me to better understand my students’ learning performance	(Tsai et al., 2021a)
15. The teaching staff will have an obligation to act (i.e., support students) if the analytics show that a student is at-risk of failing, underperforming, or that they could improve their learning	(Tsai et al., 2021a)

B Appendices related to methodology

See Tables 5 and 6

**Table 5:** Summary of trust of the teaching staff in LA stakeholders and the results of the paired t-tests for each item, sorted by mean difference (ideal minus predicted expectations)

Survey item	Ideal M (SD)	Pred M(SD)	Diff	Paired t-test
4. Access data about my students' progress	6.20(1.09)	5.95(0.93)	0.25	t = 1.814, p = 0.074, Cohen's d = 0.266
5. Access data about any students within a programme&	5.72(1.41)	5.30(1.24)	0.42	t = 2.402, p = 0.019, Cohen's d = 0.32
15 Staff will have an obligation to act on LA	6.03(1.17)	5.47(1.18)	0.56	t = 3.475, p = 0.001, Cohen's d = 0.47
1. Guidance on how to access learning analytics about my students&	6.19(0.92)	5.63(1.18)	0.56	t = 3.729, p = 0.000, Cohen's d = 0.28
3. Facilitate open discussions to share experience of LA services	5.72(1.38)	5.14(1.44)	0.58	t = 3.599, p = 001, Cohen's d = 0.41
7. Provide support to struggling students	6.00(1.17)	5.38(1.40)	0.62	t = 2.859, p = 0.006, Cohen's d = 0.49
2. Provide staff with opportunities for professional development	6.14(1.03)	5.45(1.23)	0.69	t = 4.936, p = 0.000, Cohen's d = 0.61
8. Update students about their learning progress	5.97(1.31)	5.21(1.48)	0.76	t = 3.301, p = 0.002, Cohen's d = 0.55

**Table 6:** Summary of the trust of teaching staff in LA tools and the result of the paired t-test, sorted by mean difference (ideal minus predicted expectations

Survey item	Ideal M (SD)	Pred M(SD)	Diff	Paired t-test
6. Allow students to make their own decisions	5.60(1.34)	5.17(1.33)	0.43	t = 2.301, p = 0.025, Cohen's d = 0.32
14. Allow me to better understand students' learning performance	6.18(1.22)	5.63(1.23)	0.55	t = 4.279, p = 0.000, Cohen's d = 0.46
9. Collect and present data that is accurate	6.00(1.15)	5.35(1.35)	0.65	t = 4.378, p = 0.000, Cohen's d = 0.52
12. Present students with a complete profile	6.14(1.06)	5.46(1.33)	0.68	t = 3.933, p = 0.000, Cohen's d = 0.56
10. Compare learning progress to goals/ course objectives	6.00(0.95)	5.14(1.47)	0.86	t = 4.668, p = 0.000, Cohen's d = 0.71
11. The feedback from the LA will be understandable	6.21(1.00)	5.33(1.47)	0.88	t = 4.173, p = 0.000, Cohen's d = 0.71
13. Promote students’ academic and professional skill development	6.15(1.03)	5.23(1.63)	0.92	t = 4.490, p = 0.000, Cohen's d = 0.7

## Footnotes

1 http://www.sheilaproject.eu/sheila-framework

2 A web-based survey software https://www.qualtrics.com/au

3 https://www.qsrinternational.com/nvivo-qualitative-data-analysis-software/home

4 MAXQDA | All-In-One Tool for Qualitative Data Analysis & Mixed Methods—MAXQDA.
